# Ghrelin in Diabetes and Metabolic Syndrome

**DOI:** 10.1155/2010/248948

**Published:** 2010-04-27

**Authors:** Leena Pulkkinen, Olavi Ukkola, Marjukka Kolehmainen, Matti Uusitupa

**Affiliations:** ^1^Department of Clinical Nutrition, Food and Health Research Centre, University of Eastern Finland, Kuopio Campus, Institute of Public Health and Clinical Nutrition, University of Eastern Finland, Campus P.O. Box 1627 FI-70211 Kuopio, Finland; ^2^Institute of Clinical Medicine, Department of Internal Medicine, Biocenter Oulu, 90014 University of Oulu and Research Center of Oulu University Hospital, Oulu, Finland

## Abstract

Metabolic syndrome is a cluster of related risk factors for cardiovascular disease, type 2 diabetes and liver disease. Obesity, which has become a global public health problem, is one of the major risk factors for development of metabolic syndrome and type 2 diabetes. Obesity is a complex disease, caused by the interplay between environmental and genetic factors. Ghrelin is one of the circulating peptides, which stimulates appetite and regulates energy balance, and thus is one of the candidate genes for obesity and T2DM. During the last years both basic research and genetic association studies have revealed association between the ghrelin gene and obesity, metabolic syndrome or type 2 diabetes

## 1. Introduction

A great deal of evidence suggests that ghrelin is involved in the development of metabolic syndrome and type 2 diabetes (T2DM). Ghrelin plays also an important role in cardiovascular system. We have examined ghrelin and its genetic variation with respect to the occurrence of the components of metabolic syndrome and the risk of T2DM. In this paper we give an overview of what is known about the role of ghrelin in obesity, insulin resistance, T2DM, and cardiovascular disease, and how ghrelin is involved in the regulation of glucose, insulin, adipose tissue, and cardiovascular metabolism. We also discuss the putative role of genetic variation in the ghrelin and ghrelin receptor genes in metabolic syndrome and T2DM. 

## 2. Ghrelin Concentrations in Obesity, Insulin Resistance, and Type 2 Diabetes Mellitus

The recent literature suggests that in addition to food intake and energy balance, ghrelin also controls glucose metabolism [1]. Furthermore, current evidence suggests that ghrelin could contribute to the metabolic syndrome [1]. It has been shown that ghrelin concentrations are reduced in different pathophysiological conditions including obesity, type 2 diabetes, and other conditions with metabolic disturbances [2, 3].

Ghrelin is a target for posttranslational modifications, which results in two different forms of circulating ghrelin: unacylated ghrelin (UAG) and acylated ghrelin (AG), in which Ser 3 is octanoylated [4]. A relative excess of AG compared to UAG has been reported in insulin resistance and related conditions [3] raising the possibility that UAG/AG ratio could play a role in development of metabolic syndrome. 

Plasma ghrelin concentration has been shown to be lower in obese Caucasians when compared with lean Caucasians [2, 3, 5, 6], and in some studies higher AG concentrations have been reported in obese but otherwise healthy subjects compared to nonobese healthy subjects [3]. In persons with type 2 diabetes the fasting ghrelin concentrations are lower in obese than in lean persons and the similar ratio is with AG concentrations [7]. Circulating ghrelin concentrations are also reduced in healthy offspring of type 2 diabetic patients [8] indicating the presence of possible genetic component in the regulation of ghrelin plasma levels. When the ghrelin concentrations were compared between lean Caucasians and lean Pima Indians, it was found that the concentration was significantly lower in Pima Indians, the population with high tendency to obesity and type 2 diabetes [5]. 

There are also differences in fasting and postprandial ghrelin concentrations in nondiabetic populations between lean and obese persons. Postprandial plasma ghrelin is suppressed proportional to meal calorie content in normal weight [9] but not in obese subjects [10], which suggest that food intake fails to suppress ghrelin levels in obese humans [11]. 

Low ghrelin concentrations are also associated with higher prevalence of the metabolic syndrome with progressively lower ghrelin levels in relation to the number of components of the metabolic syndrome [1, 12]. This is mostly explained by higher BMI in subjects with lower ghrelin levels, because adiposity influences all other features of the metabolic syndrome [1–3, 5, 12–14]. In fact, it has been shown that total plasma ghrelin as well as UAG concentrations are lower in obese patients with metabolic syndrome compared to nonobese counterparts [14]. Furthermore, among obese subjects, plasma ghrelin levels are lower in insulin resistant persons compared to insulin sensitive persons [15]. However, the concentrations of total ghrelin, AG, or UAG separately are not different between insulin sensitive and insulin resistant persons with similar body weight [1]. Among overweight and obese patients, the ratio of AG : UAG is lower in insulin sensitive than in insulin resistant subjects [3, 15]. 

Polycystic ovary syndrome (PCOS) is associated with adiposity and metabolic changes predisposing to insulin resistance and type 2 diabetes mellitus [16–18]. Obese patients with PCOS have lower levels of ghrelin than BMI matched control subjects [19], although in another study no difference was observed in this regard [20].

## 3. Effect of Weight Loss on Ghrelin Concentration

It has been suggested that ghrelin is linked to excessive food intake in two ways. Firstly, the attenuated postprandial reduction in ghrelin levels may directly increase the length of time for which the subject feels hungry, and secondly, as a consequence of the elevated ghrelin levels, the speed of gastric emptying may not be reduced, and the resulting feeling of satiety not elicited [11]. Without these feelings of satiety, obese individuals eat more than they need, and thus gain weight [13].

So far, the majority of studies have focused on the effects of diet induced or combined exercise/diet weight loss on total ghrelin concentrations [21–28]. These studies are very diverse, with different interventions, intervention periods, age and number of participants, and also inclusion criteria. Most studies have shown that weight reduction increases ghrelin concentrations in obese subjects [21–23, 29] or the concentration is unchanged in overweight healthy adults or obese children after weight loss [24]. However, during weight maintenance after the weight loss, ghrelin levels tend to decrease back to the levels they were before weight loss [25]. Furthermore, an initial decrease along with weight loss and subsequent increase in plasma ghrelin has been reported [26]. Weight loss is also shown to result in increased ghrelin concentrations in normal weight individuals [27]. Only a few studies have been conducted to investigate the exclusive effect of weight loss through exercise intervention on plasma ghrelin levels. In general, these studies have shown either an increase [28] or no change on ghrelin concentrations [30].

## 4. Effect of Insulin and Glucose Concentrations on Ghrelin Secretion

Insulin is shown to inhibit ghrelin secretion in healthy normal-weight and overweight persons [15, 31, 32], and both oral and intravenous glucose loads are also shown to regulate ghrelin secretion in humans [33–37]. Insulin and HOMA-IR are associated negatively with total ghrelin and UAG concentrations while AG had positive association [3]. Liu and coworkers developed recently a new reliable sandwich method for detection of AG and UAG separately. With this new assay they showed evidence that ghrelin acylation and secretion are regulated separately. The use of this method may facilitate more reliable detection of different ghrelin forms in future [38]. Physiological increases in insulin levels may play a key role in regulating postprandial plasma ghrelin concentrations, since meal-induced ghrelin suppression is absent in severe insulin deficiency [10]. An increase in insulin after the oral or intravenous glucose administration could contribute to the inhibitory effect of glucose on ghrelin concentrations. However, the administration of a combined pulse of glucose and insulin does not acutely suppress ghrelin levels [36–39]. Reduction in ghrelin after intravenous glucose bolus in subjects with type 2 diabetes suggests that early insulin response does not affect plasma ghrelin [39]. Indeed, during euglycaemic clamp, an increase in insulin levels leads to suppression of ghrelin levels and is remained suppressed during subsequent hypoglycemia and even fell further during following hyperglycaemia. However, another study has shown that hyperinsulinaemia with concomitant hyperglycaemia at concentrations typically seen in insulin-resistant subjects does not affect plasma ghrelin but is decreased only at pharmacological insulin concentrations [40]. However, it is still unclear whether insulin and glucose per se play a direct inhibitory role in ghrelin secretion [16]. The decrease in ghrelin levels after an oral glucose load is modulated by sex, status of obesity, and level of insulin resistance [41]. Ghrelin concentrations are shown to be higher in women than in men. To support of this finding, ghrelin concentrations are shown to correlate positively with testosterone concentrations [41].

## 5. Effect of Ghrelin on Glucose and Insulin Metabolism

Above we discussed the associations of ghrelin with glucose and insulin levels. However, ghrelin may also participate in the regulation of glucose and insulin metabolism as discussed in this section in more detail. Because glucose and insulin metabolism are tightly connected, their effects are difficult to separate from each other.

 A human study conducted recently in morbidly obese nondiabetic persons showed that administration of combination of AG and UAG reduced insulin concentrations significantly without any effect on glucose concentrations, while AG or UAG alone did not have any significant effects [42]. Based on this data, it was concluded that insulin sensitivity was improved in these persons [42]. It has been shown that specifically AG is responsible for improving insulin sensitivity, while UAG has opposite effects [43]. However, in another study conducted with healthy young persons showed that administration of ghrelin impairs insulin and glucose metabolism by increasing glucose concentrations and decreasing insulin levels [44], and a number of other studies have supported these findings [45–48] 

Acute ghrelin administration in humans increases plasma glucose levels by downregulation of insulin, and arginine is shown to amplify the hyperglycaemic effect of ghrelin which can be blocked by the administration of the GHS-R agonist D-(Lys^3^)-GHRP6. To support these findings, plasma ghrelin levels are shown to correlate negatively with insulin concentrations and are associated with fasting insulin levels, insulin resistance, and obesity [49, 50]. Specifically, AG is shown to be responsible for the decrease in insulin and a consequent rise in glucose levels [49]. It has been suggested that UAG alone has been suggested to be devoid of any endocrine effects but is able to antagonize the effects of AG on insulin secretion [51]. There is evidence that UAG has a specific functional role in insulin signaling since it has been shown to stimulate insulin secretion in pancreatic cell lines [52, 53]. Furthermore, the combination of AG and UAG may improve insulin sensitivity [51]. This has been shown in GH-deficient patients, in whom UAG prevents the rapid rise in insulin and glucose levels when coadministered with AG [51]. The hyperglycaemic effect of ghrelin could be mediated through activation of catecholamine-induced glycogenolysis or directly by acting on hepatocytes; where it may enhance gluconeogenesis [45, 54]. Interestingly, AG has been shown to stimulate glucose output by primary hepatocytes; whereas UAG mediates an inhibitory effect [49]. Moreover, it counteracts the stimulatory effect of AG on glucose release [49]. 

Ghrelin is shown to be expressed in pancreas both in rodents and humans [55–57], where it may locally modulate insulin secretion. These findings suggest that ghrelin has a pathophysiological role in regulation of insulin release. Ghrelin is found to inhibit insulin release in rodents and in isolated islets in vitro [58–60], to promote survival of both INS-1E *β* cell line and human islets of Langerhans [52] and to stimulate insulin secretion in pancreatic cell lines [52, 53]. Furthermore, ghrelin is shown to prevent cell death and apoptosis of HIT-T15 pancreatic *β* cell line [60]. Ghrelin treatment of neonatal rats exposed to streptozotocin attenuates the development of diabetes and is associated with increased islet neogenesis, suggesting that ghrelin might have a proliferative or cytoprotective effect on *β* cells [60]. In mice, ghrelin has also shown to hamper insulin's capacity to suppress endogenous glucose production; whereas it reinforces the action of insulin on glucose disposal [61]. Furthermore, simultaneous administration of UAG abolishes the inhibitory effect of ghrelin on hepatic insulin action [61].

## 6. Effect of Ghrelin on Adipose Tissue

Adipose tissue is one of the most important organs mediating metabolic effects by numerous adipokines and cytokines, which are secreted from adipose tissue [62]. There is increasing amount of evidence that also ghrelin may have an important role in modulating function of adipose tissue. Because obesity has a significant role in modulating the expression of ghrelin, it is important to know how ghrelin is involved in the regulation of adipocyte metabolism. Several studies have suggested that ghrelin may play an important role in adipogenesis and storage of energy in adipose tissue [63–65]. Chronic ghrelin administration has been shown to increase body fat content in rodents and humans [63]. In visceral adipose tissue, ghrelin (AG and UAG) is shown to stimulate lipid accumulation by enhancing the expression of adipogenic genes including PPARg, SREBP1, acetyl-CoA carboxylase, fatty acid synthase lipoprotein lipase, perilipin, adipocyte determination and differentiation-dependent factor (ADD)1, and adipose protein 2/fatty acid binding protein (aP2) during adipocyte differentiation [66]. These functions might be mediated via AMPK pathway [67]. It has been demonstrated that infusion AG and UAG simultaneously in rats independently modulates adipocyte metabolism by inhibiting isoproterenol induced lipolysis [68], regulating adipogenesis [69, 70], suppressing noradrenalin release in brown adipose tissue [71], and promoting glucose and triglyceride uptake and antiapoptotic actions [65]. Ghrelin is also shown to stimulate lipogenesis and to inhibit lipid oxidation in white adipocytes; whereas in brown adipocytes central ghrelin infusion results in decreased expression of uncoupling proteins, molecules contributing to energy dissipation [69]. All of these findings strongly support the view that ghrelin may have an “energy saving” effects on adipose tissue. 

In addition, Ghrelin has also been shown to stimulate adipogenesis in vitro [70, 93], and both AG and UAG directly promote bone marrow adipogenesis in vivo [70, 93]. However, Zang et al. have shown that ghrelin inhibits adipogenesis by stimulating cell proliferation in mouse adipocyte cell line [94]. Ghrelin also inhibits the expression of adiponectin. It is of note that the reduced concentrations of adiponectin have been implicated in the pathogenesis of insulin resistance and obesity [95]. Furthermore, ghrelin exerts a receptor-mediated stimulatory effect on leptin production of cultured rat white adipocytes [96].

## 7. Ghrelin and Immunomodulation

Given the wide distribution of functional GHSR on various immune cells, it was hypothesized that ghrelin may exert immunoregulatory effect on immune cell subpopulations [97]. In vitro, ghrelin treatment is shown to inhibit production of proinflammatory cytokines (interleukin IL1*β*, IL6, and TNF*α* by PBMCs via a GHSR-specific pathway [97]). It was further reported that ghrelin inhibits IL6 and TNF*α* mRNA expression in primary human T cells, which suggests a role for ghrelin in the transcriptional regulation of inflammatory cytokine expression [98]. 

## 8. Effects of Ghrelin on Cardiovascular System

Ghrelin has diverse cardiovascular effects, which are most probably ghrelin receptor mediated rather than GH mediated, since expression of ghrelin receptor has been reported in the cardiovascular system [99]. Administration of ghrelin in persons with metabolic syndrome is shown to improve endothelial function by preventing proatherogenic changes [100] and improving vasodilatation [101], by decreasing blood pressure (BP) without an increase in heart rate [102], and additional haemodynamic effects by increasing cardiac output [103]. Chronic subcutaneous administration of ghrelin in rats is shown to exert a therapeutic effect in heart failure by improving left ventricular dysfunction and attenuation of the development of cardiac cachexia [104], by improving left ventricular dysfunction and attenuating the development of left ventricular remodeling and cardiac cachexia in rats with CHF [105]. Plasma ghrelin concentrations are shown to correlate positively with carotid artery atherosclerosis [106]. In addition, ghrelin receptor is upregulated in heart muscle of patients suffering from end-stage heart failure [106]. 

Molecular mechanisms for the cardiovascular activity of ghrelin have been intensively studied in cell culture models [107, 108]. It has been demonstrated that ghrelin stimulates nitric oxide (NO) production both in cultured endothelial cells and in intact vessels [107, 108], while the NO synthesis can be blocked by NO synthase inhibitor (NOS) (*N* ^*G*^ nitro-L-arginine methyl ester), by phosphatidylinositol 3-kinase inhibitor (wortmannin) or by antagonist of ghrelin receptor (D-Lys^3^) [107]. Furthermore, ghrelin is shown to mediate NO production through phosphorylation of endothelial nitric oxide synthase (eNOS) [108], Akt, one of the main kinases involved also in insulin signaling pathway [107, 108] and AMP-activated protein kinase (AMPK), in endothelial cells and in intact vessels [108]. Based on these findings ghrelin uses partly insulin signaling pathway for production of NO. Furthermore, downregulation of GHSR-1 by siRNA blocks the NO production and phosphorylation of Akt and endothelial NOS indicating that these functions are mediated by GHSR-1 [107].

Togliatto and coworkers [109] studied separately the effects of AG and UAG on mobilization of endothelial progenitor cells (EPCs) in healthy humans, persons with T2DM, and in ob/ob mice. They found that the treatments had no effect in healthy human subjects. However systemic administration of UAG but not AG prevented diabetes-induced EPC damage by modulating the NAPDH oxidase regulatory protein Rac1 and improved their vasculogenic potential both in individuals with T2DM and ob/ob mice [109]. UAG also facilitated the recovery of mobilization of EPC. Crucial to EPC mobilization by UAG was the rescue of NO synthase phosphorylation by Akt. Furthermore, EPCs expressed UAG binding sites, which were not recognized by AG [108].

To support earlier findings above, Tesauro and coworkers [110] conducted a human study in persons with obesity and metabolic syndrome in order to test if exogenous ghrelin could improve the balance between NO and endothelin-1, a vasoconstrictor peptide produced by vascular endothelial cells. In the absence of ghrelin, the vasodilator response to BQ-123, an endothelin A receptor antagonist, was greater in patients than in controls; whereas infusion of NO synthase inhibitor induced smaller vasoconstriction in patients than in controls [110]. Exogenous ghrelin decreased the vasodilator response to BQ-123 and enhanced the magnitude of changes in forearm blood flow induced by NO synthase inhibitor in patients but not in controls [110]. The favorable effect of ghrelin on endothelin A-dependent vasoconstriction was likely related to the stimulation of NO production, because no change in the vascular effect of BQ-123 was observed after ghrelin in persons with metabolic syndrome during continuous infusion of the NO donor sodium nitroprusside. In patients with metabolic syndrome, ghrelin has benefits to normalize the balance between vasoconstrictor (endothelin 1) and vasodilating (NO) mediators, thus suggesting that this peptide has important peripheral actions to preserve vascular homeostasis in humans [110].

## 9. Ghrelin O-Acetyltransferase (GOAT)

The peptide hormone ghrelin is the only known protein modified with an O-linked octanoyl side group, which occurs on its third serine residue. This modification is crucial for ghrelin's physiological effects including regulation of feeding, adiposity, and insulin secretion [4]. It is no longer than two years ago when an enzyme ghrelin O-acyltransferase (GOAT), which links octanoate to Ser3 of ghrelin, was discovered by two different research groups [111, 112]. Human GOAT is able to acylate ghrelin also with other fatty acids, besides octanoate, ranging from acetate to tetradecanoid acid [112]. Analysis of the mouse genome revealed that GOAT belongs to a family of 16 hydrophobic membrane-bound acyltransferases and is the only member of this family that octanoylates Ser3 position of ghrelin peptide when coexpressed in cultured endocrine cell lines with prepro-ghrelin [111, 112]. Expression levels of gastric GOAT are the highest under ad libitum and are decreased with fasting, showing similar pattern of decrease to that of ghrelin [112]. GOAT expression is localized in ghrelin producing cells in gastric mucosa [112–114] as well as in pancreas [111, 112]. It has been found that the genetic disruption of the GOAT gene in mice leads to complete absence of AG in circulation [112]. 

Kirchner and coworkers have recently studied the role of GOAT in regulating of the activity of ghrelin using different animal models [115]. They showed that GOAT functions as a gastric lipid sensor linking selected ingested nutrients with hypothalamic energy balance regulation via endocrine ghrelin system [115]. Animal models have shown that GOAT is required and sufficient to mediate the impact of dietary lipids on body adiposity, and that activation of the GOAT-ghrelin system is triggered by a lipid-rich environment rather than by caloric depreviation [115]. Specifically, sufficient dietary supply of medium chain triglycerides is important for ghrelin acylation [115].

The discovery of GOAT has provided possibilities to develop tools to study specific functional roles of the two different ghrelin forms, UAG and AG, in human health in more detail. For example, modification of its expression provides tools to study the function of different ghrelin forms and makes possible to develop drugs against obesity and related conditions.

## 10. Therapeutic Potential against Obesity and Insulin Resistance by Targeting GOAT/Ghrelin System

Increasing prevalence of obesity throughout the world is becoming an increasing health burden. Because obesity is a strong risk factor for development of cardiovascular diseases and T2DM, the development of strategies to combat obesity epidemic is urgently needed. AG and UAG forms of ghrelin as well as GOAT are attractive targets to develop pharmacological treatments for obesity and diabetes. 

Pharmaceutical companies have started actively to develop drugs that can target orexigenic or obesity related functions of ghrelin, its receptor, or GOAT [116–119]. Ghrelin receptor antagonists are shown to block GH secretion and thus improve the diabetic condition by promoting glucose-dependent insulin secretion and weight loss and suppressing appetite [120]. Peptide inverse agonist DLys3-GHRP6, which blocks GHRP induced GH secretion, is shown to reduce food intake and body weight. Furthermore, vaccination of mature rats or mouse with ghrelin immunoconjugates against AG decreases feed efficiency, adiposity, and body weight gain in relation to immune response elicited against AG [121, 122]. Recently, a new class of L-RNA-based hormone antagonists, the spiegelmers (SPMs), has been developed [123]. SPMs are L-isomer oligonucleotides that are stable in biological fluids, enabling long-lasting peptide neutralization after a single application [123]. This makes these compounds very useful for experimental purposes and possibly as therapeutic agents. Unlike classic hormone antagonists, SPMs do not interact with the receptor but bind with high affinity to their target molecule and prevent binding to the endogenous receptor. The antighrelin Spiegelmer NOX-B11-3 neutralizes the stimulatory effects on GH release and food intake in animal studies [124].

## 11. Genetic Association Studies of the Ghrelin and Ghrelin Receptor Genes

Several genome-wide scans have suggested that certain areas of the chromosome 3, the same chromosome where ghrelin and ghrelin receptor genes are located, might be linked with obesity or metabolic syndrome [72, 73]. Polymorphisms in the human GHRL gene and the 5′ flanking region have been intensively studied. The most studied exonic SNPs include the Leu72Met located in exon 3 and Arg51Gln, which is located in exon 3 within the last codon of the mature ghrelin protein and disrupts the recognition site of the endoprotease, leading to proteolytic cleavage of the carboxy-terminal 66 amino acids to produce mature ghrelin [74], Table 1. Most of the association studies are focused on metabolic syndrome and T2DM, which are summarized in Table 2. A number of studies have shown associations between GHRL SNPs and obesity or related traits, although the results are contradictory (see Table 2). The Met72 allele of GHRL has been associated with earlier age at onset of obesity and higher BMI [6, 74, 78, 87, 88, 91, 125, 126], but negative findings have also been reported [6, 74, 77, 78, 85, 89]. The −501A>C in the promoter region of the GHRL gene and the intronic +3056T>C polymorphisms has been shown to associate with obesity and related conditions [79, 81], while some studies have failed to find association with these SNPs [6, 79, 81, 85, 92, 127–129]. 

In addition, ghrelin variations are also shown also to be associated with blood pressure [129]. 

Regarding the genetic association studies of *GHSR *SNPs, only a few studies have been reported so far. From these reports at least two have shown an association between GHSR SNPs and features of metabolic syndrome [75, 76], but most of the studies have shown negative results.

## 12. Take-Home Message

In terms of obesity, metabolic syndrome, and T2DM, ghrelin is very interesting hormone, which plays a crucial role in glucose and insulin metabolism and in development of obesity and insulin resistance. The knowledge on functions of ghrelin in peripheral tissues, such as pancreas, adipose, and vascular tissues has increased during the last few years. The recent discovery, the characterization of ghrelin-O-acyltransferase, GOAT has provided new challenges to develop drugs against obesity and T2D. The modification of GOAT expression provides tools to regulate the AG : UAG ratio and to study the specific roles of different ghrelin forms (AG and UAG) separately in human health. Regarding the positive cardiovascular effects of ghrelin, it is considered as a direct target for prevention of CVD. 

Regarding the genetics of ghrelin and its receptors, more studies are needed to show whether and to what extent they are involved in the pathogenesis of metabolic syndrome and T2DM. In Genome wide association studies no confirmation has been achieved in this regard.

## Figures and Tables

**Table 1:** Common names of *GHRL* SNPs with their corresponding rs-numbers.

NCBI RefSNP accession ID	Position	SNP location
rs1629816	−4427G>A	Promoter
rs3755777	−1500C>G	Promoter
rs26311	−1062G>C	Promoter
rs26312	−994C>T	Promoter
rs27647	−604G/A	Promoter
rs26802	−501A/C	Promoter
rs696217	Leu72Met	Exon 3
rs2075356	3056T>C	Intron 3
rs4684677	Gln90Leu	Exon 4
rs35684	5179A>G	3′ region
rs2072578	9344G>A	3′ region

**Table 2:** Associations of polymorphisms in the *GHRL* gene.

SNP	Risk allele	Association	Subjects	References
Leu72Met	Met72	Lower age of onset of self-reported obesity	96 obese and 96 normal-weight Swedish women	[72]

Leu72Met	Met72	Higher frequency in Whites than in Blackslower BMI, fat mass, visceral fat, total TG and RQ; higher IGF-1 levels in Blacks	784 French-Canadian subjects (Quebec Family Study) 778 subjects (276 Blacks and 502 Whites; HERITAGE Family Study)	[73]
Arg51Gln	Gln51	Not observed among Blacks	1442 subjects (741 from obese registry, 701 from normal reference population; SOS)	

Leu72Met	Met72	Higher BMI, earlier age of onset of obesity and reduced first phase insulin secretion	70 tall and obese children	[74]

Gln90Leu	Gln90	Higher frequency in obese children, but also in underweight students	215 extremely obese German children and adolescents, 93 normal-weight students,134 underweight students, 44 normal-weight adults	[75]

Leu72Met	Met72	Lower serum creatinine and lipoprotein a levels	258 Finnish Caucasians with T2D and 522 controls	[76]

Arg51Gln	Gln51	Risk allele for hypertension and T2D; predictor of 2-h plasma glucose in OGTT; lower IGF-1 and higher IGFBP-1 concentrations in normotensives; lower AUC insulin	519 hypertensive and 526 normotensive Finnish Caucasians	[77]

Leu72Met	Met72	In obese/overweight: higher neonatal weight-for-age; earlier age at onset of obesity; higher IGF-1 concentration.	81 obese or overweight and 96 normal-weight Italian children and adolescents and 72 normal-weight young adults	[6]

4427G>A	G	Diffuse large cell lymphoma	684 healthy controls and 308 North American subjects with non-Hodgkin Lymphoma	[78]

Arg51Gln	Gln51	Lower MetS frequencyHigher fasting glucose,TG, and frequency of MetS and lower HDL cholesterol	856 Old Order Amish from US	[79]
Leu72Met	Met72			

Leu72Met	Met72	More depressed and anxious in patients with methamphetamine dependence. No association with methamphetamine dependence	118 Koreans with methamphetamine dependence, 144 controls	[80]

Leu72Met		No association with obesity	222 obese Korean children	[81]

−1500C>G −1062G>C −994C>T Leu72Met	C	Lower HDL cholesterol All four SNPs: no association with T2D	760 T2D and 641 nondiabetic Koreans	[82]

Leu72Met	Leu72	Higher TG, fasting insulin and HOMA-IR. Higher fasting insulin and HOMA-IR	1420 Caucasians (500 normal weight and 920 overweight/obese)	[83]
−604>C/T	C			

Leu72Met	Met72	Lower allele frequency in diabetic nephropathy with renal dysfunction. Lower total cholesterol levels in patients with diabetic nephropathy with renal dysfunction	138 subjects with diabetic nephropathy, 69 diabetics without nephropathy	[84]

Leu72Met	Met72	Lower creatinine levels in diabetic group. No association with T2D	206 T2D, 80 controls	[28]

−501A>C	A	Higher BMI	1045 Finnish subjects from the Oulu Project Elucidating Risk for Atherosclerosis (OPERA) study	[85]

Leu72Met		No association with weight loss	771 obese Caucasian Europeans	[86]

Leu72Met	Met72	Higher allele frequency in higher BMI group than in normal-weight group. Higher BMI, waist circumference, and change in body weight from age 18	2238 middle-aged and older Japanese people	[87]
Leu72Met	Met72	Lower BMI in CAD patients but no association with CAD, no association with hypertension, T2D, or dyslipidaemia	317 Chinese CAD patients, 323 controls	[88]

Leu72Met	Met72	Higher scores on Drive for Thinness-Body Dissatisfaction subscale	264 Japanese women	[89]
3056T > *C*	C	Higher weight, BMI, fat mass, waist circumference, sum of skinfold thicknesses, self-reported past min and max BMIs and lower HDL chol		

Arg51Gln Leu72Met	Gln51	Higher cholesterol levels over time. Subjects with Gln51and /or Met72 lost body weight faster than patients with Arg51/Leu72	210 hemodialysed patients prospectively followed up to 15 months	[90]

Leu72Met	Met72	Persons with 72Met allele have lower risk to develop T2DM	507 persons with IGT: the Finnish diabetes prevention study	[91]

−604G/A	G	Persons with the most common genotype combination of the SNPs 604G/-501A/, Leu72/GLN90 have significantly lower systolic and diastolic blood pressure at baseline and during the 3-year follow-up	507 persons with IGT: the Finnish diabetes prevention study	[92]
−501A/C	A			
Leu72Met	Leu72			
GLN90Leu	GLN90			
